# The Evolving Landscape of Viral Infections in Immunocompromised Hosts

**DOI:** 10.3390/v17060787

**Published:** 2025-05-30

**Authors:** Ralph Tayyar, Dora Y. Ho

**Affiliations:** 1 Division of Allergy and Infectious Diseases, University of Washington, Seattle, WA 98195, USA; 2 Division of Infectious Diseases & Geographic Medicine, Stanford University School of Medicine, Stanford, CA 94305, USA

There has arguably never been a more dynamic and challenging time to be a clinician or researcher in the field of transplant and immunocompromised infectious diseases. Advances in immunosuppressive therapies, transplantation medicine, and cellular therapies have transformed outcomes for patients with hematologic or solid malignancies and autoimmune diseases, as well as those with hematopoietic stem cell or solid organ transplants. However, these advancements come with an evolving spectrum of viral infections that demand our continued vigilance and innovation in diagnostics, treatment, and prevention strategies. The rise of chimeric antigen receptor (CAR) T-cell therapy and bispecific antibodies has also introduced novel infection complications, including herpesvirus reactivations, and underscores the need for updated surveillance and prophylactic strategies. 

Despite overall improvements in infection-related mortality, viral infections remain a major cause of morbidity and excess healthcare costs in immunocompromised patients. Reactivation of latent viruses, such as herpesviruses and polyomaviruses, along with episodic infections from respiratory and gastrointestinal viruses, presents persistent challenges, while emerging infections, like those from arboviruses, further complicate the clinical picture. The use of advanced molecular diagnostics has improved our ability to detect viral reactivation early but also introduces challenges in interpretation, particularly when low-level viremia or DNAemia does not correlate with disease. In addition, antiviral resistance is increasingly reported, particularly with prolonged prophylaxis or treatment in transplant recipients, necessitating the development and evaluation of newer agents.

In this Special Issue of *Viruses*, we bring together a multidisciplinary group of experts to explore the latest developments in the prevention, diagnosis, and management of viral infections in immunocompromised hosts. Several key themes emerge from the contributions within this issue: 

**Optimizing and Expanding Antiviral Therapies:** A comparative study evaluating maribavir and foscarnet for CMV treatment in transplant recipients highlights the importance of balancing efficacy with toxicity, particularly nephrotoxicity. Another study investigates the impact of CMV infection on renal function in liver transplant recipients, emphasizing that viral infection may exacerbate chronic kidney disease and negatively affect long-term graft survival. Additionally, a review of adenovirus infection in transplant recipients outlines current and emerging antiviral strategies, including brincidofovir and adoptive T-cell therapy. These findings underscore the need for early diagnosis, individualized treatment, and continued innovation in antiviral drug development for high-risk viral pathogens.

**Expanding the Scope of Antiviral Stewardship:** A comprehensive review outlines principles of antiviral stewardship, focusing on opportunities to individualize therapy and reduce unnecessary antiviral use. Stewardship interventions are particularly critical in managing CMV, EBV, and respiratory viruses in transplant recipients to prevent toxicity, resistance, and excess healthcare costs.

**Emerging Challenges with Cellular Therapies and Transplant Inflammation:** A focused review of herpesvirus infections following CAR T-cell therapy and bispecific antibodies details reactivation risks, particularly for HSV, VZV, and CMV, and highlights the importance of antiviral prophylaxis and the limitations of current surveillance approaches in this population. Separately, a study of cytokine dynamics in solid organ transplant recipients explores how the post-transplant inflammatory milieu influences herpesvirus reactivations. These insights suggest a need for integrated immune monitoring to anticipate and manage viral complications across different transplant modalities.

**CMV in Non-Transplant Immunosuppressed Hosts:** A detailed review of CMV colitis in inflammatory bowel disease (IBD) patients underscores the diagnostic and therapeutic challenges in differentiating between CMV disease and IBD flares. The role of tissue pathology and immunohistochemistry is emphasized, as well as antiviral strategies for steroid-refractory ulcerative colitis.

**Innovations in Diagnostics:** Novel biomarkers such as CMV cell-mediated immunity assays and viral metagenomics are discussed across several studies, suggesting a future in which risk stratification and treatment decisions will be increasingly personalized.

**Viral Infections and Transplant Outcomes:** Arbovirus transmission through transplantation, alongside the impact of respiratory viral infections on lung transplant allograft rejection and donor-specific antibody formation, is examined. A comprehensive review also highlights the risks of hepatitis B virus reactivation in liver transplant recipients. Together, these studies underscore the need for integrated infection control strategies, careful donor screening, and vigilant post-transplant monitoring.

The research presented in this Special Issue reflects the dedication and collaborative efforts of experts in virology, immunology, gastroenterology, and transplant medicine. Their work not only deepens our understanding of viral infections in immunocompromised patients but also highlights the urgency of refining diagnostic thresholds, validating new treatment options, and implementing patient-centered stewardship strategies. We extend our sincere gratitude to the authors for their valuable contributions, to the editorial team at *Viruses* for supporting this initiative, and most importantly to the patients and their caregivers, whose experiences fuel our commitment to advancing the science of viral infection management in vulnerable populations.

